# Evaluation of Relative Frequency of Pulmonary Hypoplasia and Various Anomalies Associated with Pulmonary Hypoplasia in Fetal Autopsy Study

**DOI:** 10.5146/tjpath.2022.01594

**Published:** 2023-09-15

**Authors:** Aparna Sajjan, Surekha U Arakeri, Subhashchandra Mudanur

**Affiliations:** Department of Pathology, B.L.D.E. (Deemed to be University), Shri B.M.Patil Medical College Hospital and Research Centre, Karnataka, India

**Keywords:** Fetal autopsy, Lung weight to body weight (LW:BW) ratio, Pulmonary hypoplasia (PH), Radial alveolar count (RAC)

## Abstract

***Objective:*** Pulmonary hypoplasia (PH) is one of the commonest causes of neonatal morbidity and mortality. The suggested diagnostic criteria for PH are the Lung Weight:Body Weight (LW:BW) ratio ≤ 0.012 and/or Radial Alveolar Count (RAC) ≤ 4.1. The present study was done to determine the relative frequency of PH in fetal autopsy study by the LW:BW ratio and RAC along with evaluation of the defects/anomalies associated with PH.

***Material and Methods:*** A prospective observational study was done on fetal autopsy specimens in the Department of Pathology. Examination and grossing were done as per the standard format of fetal autopsy study. Evaluation of PH was done using the LW:BW ratio and RAC. Diagnostic criteria for PH were taken as LW:BW ratio <0.012 and/ or RAC < 4.1. Chi-square test, Student T test and Kruskal Wallis test were used in statistical analysis.

***Results:*** A diagnosis of PH was made in 45 cases. Concordance between the LW:BW ratio and RAC was observed in 33 cases amounting to 73.33%. The mean LW:BW ratio was the lowest in oligohydramnios. The mean RAC was the lowest in congenital cystic adenomatoid malformation.

***Conclusion:*** A diagnosis of PH was rendered in a greater number of cases when evaluation was done by considering both the LW:BW ratio and RAC. Hence, evaluation by both the LW:BW ratio and RAC provides a reliable index of lung growth and should be an essential part of fetal autopsy study.

## INTRODUCTION

Pulmonary hypoplasia (PH) is one of the commonest causes of neonatal morbidity and mortality (1). The incidence of PH is 9 to 11 per 10000 in the general population and its reported prevalence in perinatal autopsies is 7.8% to 26% (2,3).

PH is defined as unilateral or bilateral defective or incomplete development of lung parenchyma, airway, and vessels or incomplete development of the lung which is not appropriate for gestational age (1–3). Pathologically, lung hypoplasia is defined as a decrease in the Lung Weight to Body Weight (LW:BW) ratio and reduced Radial Alveolar Count (RAC) based on findings of autopsy examination (3). The LW:BW ratio is the consistent and better method of diagnosing PH (2). The lower limit for LW:BW ratio for fetuses older than or equal to 28 weeks is 0.012. The lower limit of LW:BW ratio in fetuses <28 weeks of gestation is 0.015 (3). PH is also diagnosed by RAC. RAC can be a reliable criterion for PH (4). RAC is measured by the number of alveoli that are traversed by a perpendicular line drawn from the center of a respiratory bronchiole to the nearest connective tissue septum (4).

PH may be primary or secondary. Primary PH is rare as compared to secondary PH. The exact etiology is not known in primary PH. However, deficiency of Vitamin A and viral infection during pregnancy are considered as possible etiological conditions. Genetic or iatrogenic factors are also mentioned as possible causes for primary PH (5). Secondary PH can occur due to abnormalities in the thoracic cavity, heart, kidneys, abnormal fetal breathing movements, and decrease in the volume of amniotic fluid (1,2). In the majority of cases, PH is the result of the reduction in thoracic volume, followed by reduced production of amniotic fluid due to renal anomalies (1).

Fetal autopsy study may help to evaluate the relative frequency of PH by evaluating the LW:BW ratio and RAC. Evaluation of PH may provide an explanation for loss of pregnancy. Hence this study was undertaken to evaluate the frequency of PH by the LW:BW ratio and RAC and also to determine the various conditions/anomalies associated with PH.

## MATERIALS and METHODS

A prospective observational study was done on fetal autopsy specimens sent to the histopathology section of the Department of Pathology of our institute from 01 December 2019 to 31 July 2021.

### Method of Collection of Data

Fetal autopsy specimens of intrauterine death (IUD), still births, and neonatal deaths sent to histopathology section of the Department of Pathology were processed according to the standard protocol. Detailed obstetric history and ultrasonography (USG) findings were collected. Anthropometric data of fetus and external examination were recorded. Internal examination was done by making an I-shaped incision and en-mass dissection was done. Internal examination of all organs was done. Details of measurements, weight, gross and microscopic examination findings of various organs were noted.

Gross examination of the lung was done as per the study done by Husain and Hessel (6). Lungs were separated from the heart, thymus, and mediastinum. Trachea was cut above its bifurcation. Weight of both the lungs was recorded in fresh state. Lungs were fixed in 10% buffered formalin. After fixation, the lungs were serially sectioned in the sagittal plane and the middle slice was submitted for routine processing of the tissue. Gross examination of other organs was done as per the standard protocol and tissue bits were given from each organ and processing was done. Sections of 3-6 μ thickness were taken and Hematoxylin and Eosin-staining was done. RAC of both right and left lung was recorded by evaluating the number of alveoli cut by a line that was dropped at a right angle to the bronchial epithelium from the center of terminal respiratory bronchioles to the nearest connective tissue septum. RAC was evaluated by counting the number of alveoli in 10 high power fields in two sections taken from lung. The average was taken as RAC (4,7). Bronchioles partly lined by epithelium were also selected (8). Diagnostic criteria for PH was taken as LW:BW ratio less than 0.012 and/or RAC less than 4.1.

All the fetal autopsy specimens of IUD, stillborn and neonatal deaths that were sent to the Department of Pathology were included. Macerated foetuses with extensive autolytic changes in the lungs were excluded from the study.

### Sample Size

With anticipated prevalence of PH 7.8-26% in the study done by Pena et al. (3) the sample size is 62.

### Statistical Analysis

The data collected was entered into Microsoft Excel and then analyzed using the 20th version of the Statistical Package for Social Sciences (SPSS) for Windows. Categorical variables were presented as numbers and continuous data was presented as Mean±standard deviation and charts. The association between qualitative data was determined by applying the Chi-square test. The continuous data was compared by Student’s T-test and Kruskal-Wallis test whenever required. A p value < 0.05 was taken as significant.

## RESULTS

Total number of fetal autopsies included was 62. Detailed history and Ultrasonography findings were collected for all cases from the records available. Evaluation of PH was done in all cases by the LW:BW ratio and RAC. Evaluation of conditions and anomalies associated with PH was also done.

The majority of the fetal autopsy cases had a gestational age of 16 to 20 weeks followed by 21 to 25 weeks amounting to 27.42% vs 24.20% of the cases. The male to female ratio was 1:1, having 30 cases each of male and female. Gender ambiguity was noted in two cases.

**Figure 1:**
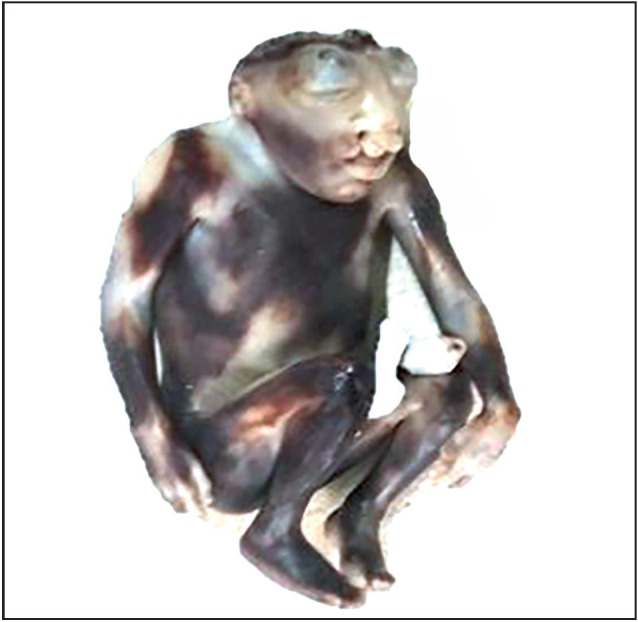
Gross photograph of fetal autopsy specimen showing neural tube defect- anencephaly with bilateral cleft lip and protrusion of eyes.

**Figure 2:**
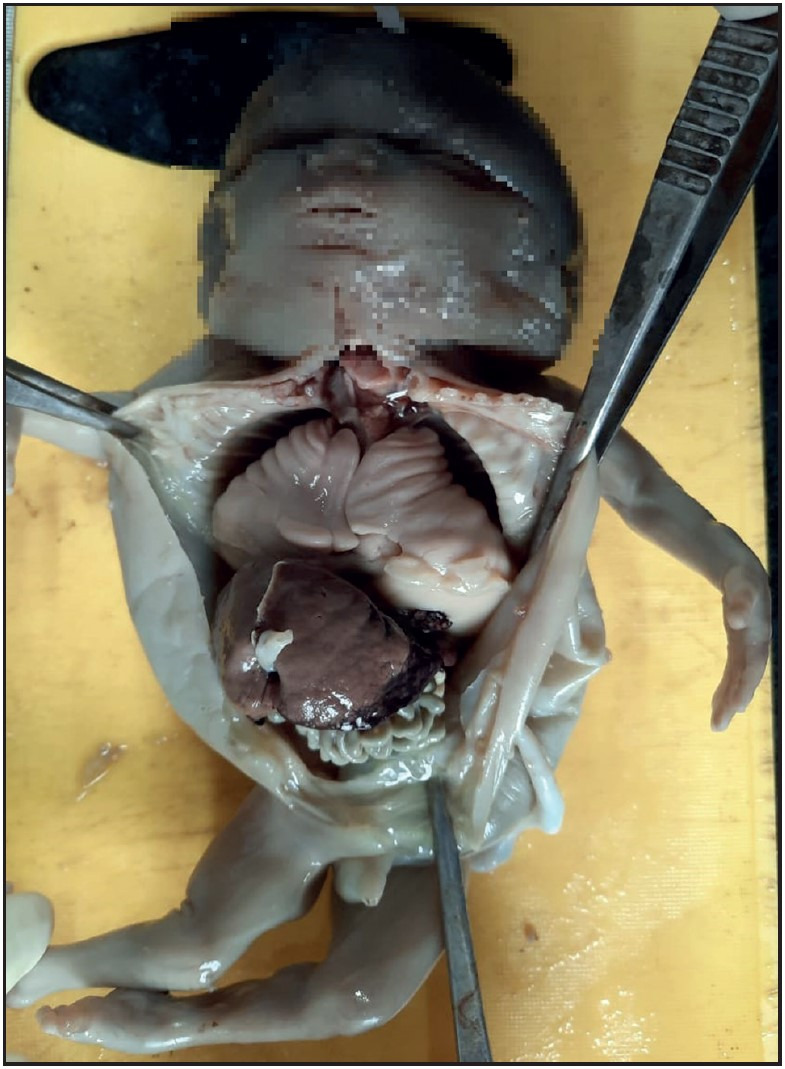
Gross photograph of autopsy specimen showing CCAM of lungs. Imprints of rib cage seen on lungs.

**Figure 3:**
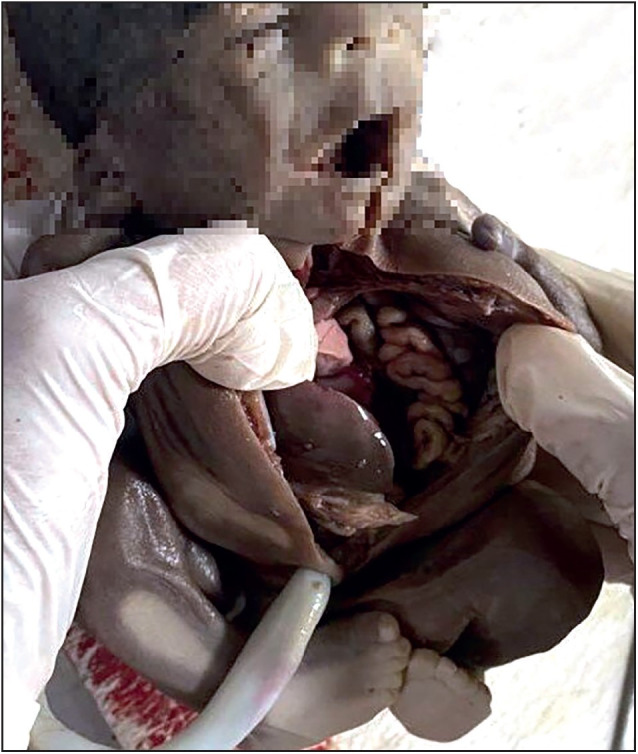
Gross photograph of CDH with intestinal loops in chest cavity.

**Figure 4:**
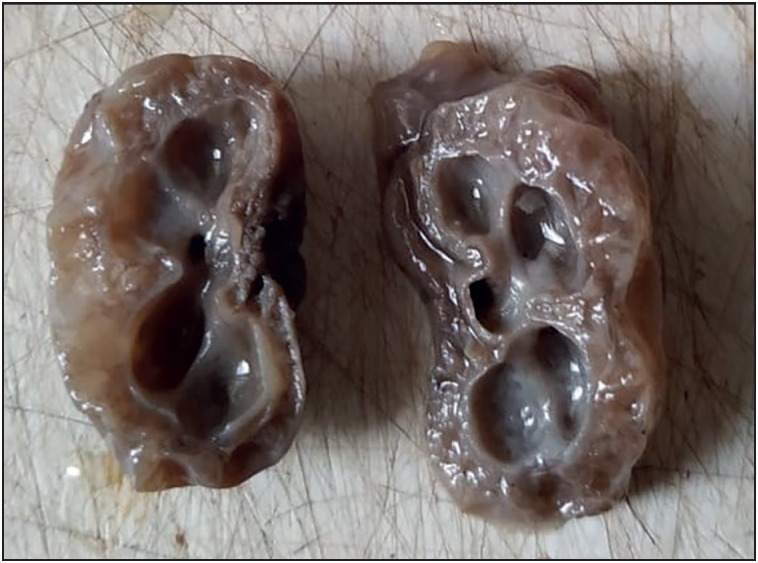
Gross photograph of bilateral multicystic renal dysplasia.

IUD was the commonest associated condition followed by oligohydramnios, neural tube defect and renal pathology. other associated anomalies were congenital cystic adenomatoid malformation (CCAM), congenital diaphragmatic hernia (CDH), premature rupture of membranes (PROM), ascites, hydrops fetalis, gastroschisis and arthrogryposis multiplex congenita (AMC) (Figure 1, Figure 2, Figure 3, Figure 4).

Categorization of PH was done based on the LW:BW ratio into four groups. In 18 cases amounting to 29.03%, the LW:BW ratio was less than and/or equal to 0.009. These cases were diagnosed as PH. In 19 cases (30.65%), the LW:BW ratio was in the range of 0.010 to 0.012. These cases were categorized as cases having a probability of PH. In 18 cases (29.03%), the LW:BW ratio was in the range of 0.013 to 0.017, where the possibility of PH was considered. In 7 cases (11.29%), the LW:BW ratio was more than and/or equal to 0.018, and in these cases PH was unlikely as per the LW:BW ratio.

The mean LW:BW ratio was highest at the GA range of 10 to 15 weeks and lowest at the GA range of 26 to 30 weeks. The mean RAC was highest at the GA range of 31 to 35 weeks, followed by 36 to 40 weeks, and was lowest at GA range of 10 to 15 weeks.

In 18 cases in which the LW:BW ratio was less than 0.009 and RAC was less than 4.1, the diagnosis was PH. In 19 cases the LW:BW ratio was between the range of 0.010 to 0.012. Out of these 19 cases, in 15 cases RAC was less than 4.1 and thus 15 cases were diagnosed as PH. However, in 4 cases RAC was higher than 4.1, and hence these cases were not categorized as PH based on RAC. In 18 cases where the LW:BW ratio was between the range of 0.013 to 0.017, in 11 cases RAC was below 4.1. Out of these 11 cases, 2 cases were of IUD cases of GA of 14 weeks. According to the review of literature the lungs are in pseudoglandular stage at less than 15 weeks of GA where developing airways resemble glands and there is no definitive lining of bronchioles (8). RAC in this age group is not conclusive. Hence, out of 11 cases only 9 cases were considered as PH. In 7 cases, the LW:BW ratio was more than 0.018 and the RAC was less than 4.1. Out of the remaining 7 cases, the GA was less than 15 weeks with lungs in the pseudo glandular stage in 4 cases. Therefore, these 4 cases were not considered as PH. Out of the 7 cases, PH was considered as per RAC in 3 cases. Thus, in 45 cases PH was considered based on the LW:BW ratio and RAC. Out of the 45 cases of PH, concordance between the LW:BW ratio and RAC was noted in 33 cases amounting to 73.33% of the cases. In these cases, RAC was less than 4.1 and the LW:BW ratio was also less than 0.012. Discordance between the LW:BW ratio and RAC was noted in 12 cases amounting to 26.66%. In these cases, RAC was less than 4.1 and the LW:BW ratio was more than 0.012. Out of these 12 cases, 4 cases were of NTD, 2 cases were of CCAM, 1 case was of gastroschisis, and 5 cases were of IUD (Table 1).

**Table 1:** Distribution of fetal autopsy cases showing concordance/discordance between LW:BW ratio and RAC.

**LW:BW**	**RAC**	**Number**	**Concordance %**	**Discordance %**
≤0.009 (n=18)	<4.1	18	100	0
	>4.1	0		
0.010-0.012 (n=19)	<4.1	15	78.94	21.06
	>4.1	4		
0.013-0.017 (n=18)	<4.1	11	61.12	38.88
	>4.1	7		
≥0.018 (n=7)	<4.1	7	0	100
	>4.1	0		

**LW:** Lung weight, **BW:** Body weight, **RAC:** Radial alveolar count.

The mean LW:BW ratio was lowest in oligohydramnios as compared to other associated conditions/anomalies. The mean LW:BW ratio and RAC was also lower in IUD and renal pathology as compared to other anomalies and the difference was statistically significant with a p value of less than 0.0001 and 0.028 respectively. RAC was lowest in CCAM as compared to other associated conditions/anomalies but only 2 cases of CCAM were noted in the present study. In neural tube defect, RAC was slightly higher than CCAM but lower than other associated conditions/anomalies, and the difference was statistically significant with p value of 0.0001 (Table 2).

**Table 2:** Association of mean gestational age, LW:BW ratio and RAC with associated conditions/anomalies.

**Cases/ Diagnosis**	**Cases** **(N=62)**	**Gestational age (mean)**	**LW:BW**			**RAC**			**P value**
			**Mean**	**SD**	**Range**	**Mean**	**SD**	**Range**	
IUD	29	25.31	0.012	0.007	0.003-0.042	3.179	1.379	0.8-5.4	<0.0001*
Oligohydramnios	8	25.87	0.008	0.004	0.002-0.014	3.455	1.325	1.65-5.4	0.0002*
Neural tube defect	8	24.25	0.020	0.010	0.010-0.037	2.262	0.435	1.7-3.2	0.0001*
Renal pathology	4	21.75	0.010	0.0005	0.010-0.011	2.437	0.675	1.65-3.0	0.0286*
Hydrops fetalis	2	30	0.023	0.020	0.006-0.042	2.56	0.847	1.7-3.9	0.3333
CCAM	2	23	0.032	0.0007	0.032-0.033	2.075	0.53	1.7-2.45	0.3333
Ascites	3	28.33	0.010	0.003	0.007-0.013	3.7	1.609	2.2-5.4	0.1000
PROM	2	29.5	0.009	0.001	0.008-0.010	3.5	2.26	1.9-5.1	0.3333
CDH	1	-	-	-	-	-	-	-	-
Gastroschisis	1	-	-	-	-	-	-	-	-
AMC	1	-	-	-	-	-	-	-	-
Uteroplacental insufficiency	1	-	-	-	-	-	-	-	-
Total	62								

*: Statistically significant (p value <0.05) **LW:** Lung weight, **BW:** Body weight, **RAC:** Radial alveolar count, **IUD:** Intrauterine death, **CCAM:** Congenital cystic adenomatoid malformation, **PROM:** Premature rupture of membranes, **CDH:** Congenital diaphragmatic hernia, **AMC:** Arthrogryposis multiplex congenita

Out of the 62 cases, meconium aspiration was noted in 23 cases amounting to 37.1%. The maximum number of cases of meconium aspiration was noted in the cases having a GA range of 31 to 35 and 36 to 40 weeks, amounting to 100% and 75% respectively. Meconium aspiration was not observed in the lung specimen of fetal autopsy cases having a gestational age less than 15 weeks. The frequency of meconium aspiration increased as the GA advanced.

## DISCUSSION

PH is congenital anomaly, caused by the arrest of normal development of the lungs during intrauterine growth. The arrest of normal development is due to insults in the thoracic cavity and extrathoracic cavities (1–3,5,9).

Askenazi and Perlman (4) mentioned that a LW:BW ratio of 0.012 and/or RAC of less than 4.1 are diagnostic criteria for PH. They also recommended that when the LW:BW ratio is less than 0.009, PH is very likely and RAC is not mandatory. When the LW:BW ratio is between 0.010-0.012, PH is probable and RAC should be indicated for confirmation. When the LW:BW ratio is between 0.013- 0.017, PH is possible and RAC is must. When LW:BW ratio is more than 0.018, PH is unlikely and RAC is not indicated. In the existing study, PH evaluation was done by the LW:BW ratio and RAC as mentioned in the study done by Askenazi and Perlman (4).

In study done by Cherian et al. (10), PH was noted in more than 10% of neonatal autopsies and they also mentioned that more than 85% of cases PH occur in association with other conditions/malformations. In the present study, out of 62 cases of fetal autopsy PH was noted in 45 cases amounting to 72.58% of cases.

In studies done by Aghabiklooei et al. (1), Wigglesworth and Desai (11), Husain and Hessel (6) and Cherian et al. (10), PH was noted in 11.3%, 14.5%, 26%, and 43% respectively. In the present study, PH was noted in 72.58%.

Out of 45 cases of PH, concordance between the LW:BW ratio and RAC was noted in 33 cases amounting to 73.33% of the cases. In these cases, RAC was less than 4.1 and the LW:BW ratio was also less than 0.012. Discordance between the LW:BW ratio and RAC was noted in 12 cases amounting to 26.66%. In these cases, RAC was less than 4.1 and the LW:BW ratio was more than 0.012. Further analysis of these cases was done to conclude the diagnosis of PH. Out of these 12 cases, 4 cases were of NTD, 2 cases were of CCAM, 1 case was of gastroschisis, and 5 cases were of IUD.

**Figure 5:**
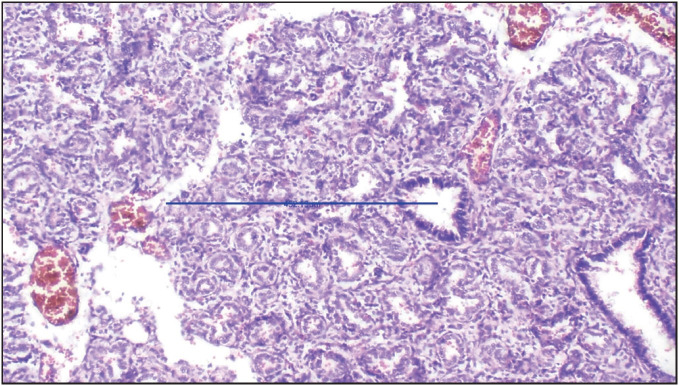
Photograph of microscopy of lung section in a case of anencephaly with mean RAC of 1.6. (H&E stain, 100x).

Askenazi and Perlman (4) observed a discrepancy between the LW:BW ratio and RAC in cases of PH with neural tube defects. They mentioned that this discrepancy could be due to a low body weight due to the absence of the brain leading to a higher LW:BW ratio and a delay in alveolar development due to absence of pituitary gland leading to a low RAC. They observed that neural tube defects had a mean GA of 38 weeks, mean LW:BW ratio of 0.016, and mean RAC of 3.1. A similar explanation holds true in our study. Six cases of neural tube defects had a mean GA of 28.1 weeks, a mean LW:BW ratio of 0.016, and a mean RAC of 2.3, which might be the reason for the discordance in the findings between the LW:BW ratio and RAC (Figure 5).

**Figure 6:**
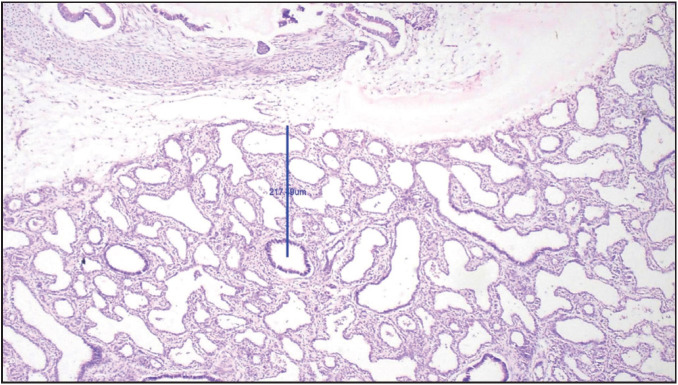
Photograph of microscopy of lung section with mean RAC of 1.7 in another case of congenital cystic adenomatoid malformation. (H&E stain, 100x)

Chikkannaiah et al. (12), in their study with 2 case reports of CCAM in the lungs mentioned that enlargement of the affected lungs and multiple cysts of varying sizes are noted in CCAM. Dos Reis et al. (13) mentioned in their autopsy findings that the affected lungs in CCAM weighed 75.9 gm in their study on CCAM with GA of 25 weeks and showed varying sized cysts with RAC less than 3. In the present study, 2 cases of CCAM of lungs were seen with mean gestational age of 23 weeks. Both cases showed enlarged lungs with one case showing imprint of the rib cage on the lung surface. In these 2 cases, the LW:BW ratio was more than 0.012 as the lung weight was increased as mentioned in studies done by Chikkannaiah et al. (12) and Dos Reis et al. (13). This might be the reason for the discordance between the LW:BW ratio and RAC findings of these cases in present study (Figure 6).

Nimrod et al. (14) found that there is greater impact on fetal growth when PROM occurred before 26 weeks of GA in their study on effect of PROM on oligohydramnios and fetal development. PROM leading to oligohydramnios syndrome consists of the tetrad of PH, skeletal deformities, Potter’s facies, and intrauterine growth retardation. Rotschild et al. (15) observed PH in 16% of fetuses that experienced oligohydramnios following rupture of membranes before 29 weeks of GA. Husain and Hessel (6) observed that 4 cases with oligohydramnios had PH with mean LW:BW ratio of 0.011 and mean RAC of 2.7 in their study. In the present study, 6 cases of oligohydramnios and one case of PROM amounting to 11.29% were considered as PH with all cases having GA of less than 29 weeks. These cases had a mean GA of 22.85 weeks, mean LW:BW ratio of 0.006, and mean RAC of 2.65, which correlated with these studies.

In the current study, 6.45% of the cases of renal pathology cases had a mean GA of 21.2 weeks, mean LW:BW ratio of 0.010, and mean RAC of 2.43. Our study findings correlate with the studies by Askenazi and Perlman (4) and Husain and Hessel (6); in their studies, they observed renal pathology cases at a mean GA of 36 weeks and 31 weeks with a mean LW:BW of 0.010 and 0.012 and mean RAC of 3.2 and 2.6, respectively.

In the present study 3.22% cases of hydrops fetalis had a mean GA of 30 weeks, mean LW:BW ratio of 0.007, and mean RAC of 3.1. Our study findings correlate with the studies by Askenazi and Perlman (4) and Husain and Hessel (6); in their studies they observed that hydrops fetalis cases had a mean GA of 34 weeks and 24 weeks, mean LW:BW of 0.013 and 0.011, and mean RAC of 4.4 and 2.2, respectively.

In the present study associated anomalies such as neural tube defect, oligohydramnios, renal pathology, lung pathology, hydrops fetalis, diaphragmatic hernia, musculoskeletal deformity, premature rupture of membranes, and gastroschisis were noted in 24 cases amounting to 53.34%. In PH, 40% cases were of IUD, 13.34% cases were of NTD, 13.34% were cases of oligohydramnios, 8.90% cases were of renal pathology, 4.44% cases each were of CCAM and hydrops fetalis, and 2.22% cases each were of PROM, gastroschisis, CDH and AMC.

Aghabiklooei et al. (1) in their study found 11.3% of primary causes of PH. Husain and Hessel (6) found 22% cases of primary PH where one third of the cases were not associated with any congenital malformations. In the current study, 18 cases of PH had no congenital malformation or other associated conditions.

In a study by Ward and Caughey (16), it was observed that the risk of meconium aspiration syndrome increases as the gestational age increases. In their study, they mentioned that meconium aspiration syndrome was observed in 1.3% at 38 weeks of GA and 4.8% at 42 weeks of GA. They also mentioned that the risk of meconium aspiration syndrome increased by 30% with each week of GA. Fischer et al. (17) also stated that the risk of meconium aspiration syndrome and meconium-stained amniotic fluid increases with advancing gestational age. The rate of meconium aspiration syndrome in their study was 0.11% at 37-38 weeks of GA, 0.20% at 39-41 weeks of GA, and 0.49% at 42-43 weeks of GA. Our study findings also correlate with the observations by these authors. In the present study, meconium aspiration was noted in 37.1% of the cases. The maximum number of cases of meconium aspiration was noted in the cases having a GA range of 31 to 35 and 36 to 40 weeks amounting to 100% and 75 % respectively.

## CONCLUSION

In the current study, PH was incidentally discovered in some clinically unsuspected cases, which may be cause of mortality. The diagnosis of PH was rendered in a greater number of cases when evaluation was done by considering both the LW:BW ratio and RAC. Thus, evaluation by both the LW:BW ratio and RAC provides a reliable index of lung growth and should be an essential part of fetal autopsy study.

## Conflict of Interest

None.
